# Plant life history strategies vary in subtropical forests with different disturbance histories: an assessment of biodiversity, biomass, and functional traits

**DOI:** 10.3389/fpls.2023.1230149

**Published:** 2024-01-10

**Authors:** Julian Liu, Haojun Xia, Zihong Zheng, Yunquan Wang, Jianhua Chen, Jian Ni, Mingjian Yu, Weicheng Zheng, Libin Liu

**Affiliations:** ^1^ The Administration Center of Zhejiang Jiulong Mountain National Nature Reserve, Lishui, China; ^2^ College of Life Sciences, Zhejiang Normal University, Jinhua, China; ^3^ College of Life Sciences, Zhejiang University, Hangzhou, China; ^4^ Suichang Ecological Forestry Development Center, Lishui, China

**Keywords:** species composition, biomass accumulation, morphological traits, adaptation strategy, disturbance history, subtropical forests

## Abstract

Disturbance alters environmental conditions in forests. Plants growing in forests with different disturbance histories in diverse environments may adopt varying life history strategies, but few studies focus on this effect. This study comprehensively investigated plant biodiversity, biomass, and functional traits in subtropical forests with two different disturbance histories in east China to explore differences in life history strategies. Biodiversity was slightly higher in disturbed compared to conserved forests. Significantly higher biomass was measured in conserved relative to disturbed evergreen broadleaved forests (*P* < 0.05). In conserved forests, leaf tissue density (LTD) was significantly higher and leaf thickness (LT), leaf dry matter content (LDMC), twig tissue density (TTD), twig dry matter content (TDMC), bark tissue density (BTD) and dry matter content (BDMC), and stem tissue density (STD) and dry matter content (SDMC) were significantly lower than in disturbed forests (*P* < 0.05). In terms of associated plant biodiversity, biomass, and functional traits, conserved forests adopted a resource acquisition strategy, reducing biodiversity and developing multiple functional traits such as high leaf area and specific leaf area and low LT, LDMC, TTD, TDMC, BTD, BDMC, STD, and SDMC to support a high biomass accumulation rate. Disturbed forests adopted a resource conservation strategy, enhancing biodiversity and developing converse trait combinations to lower the rate of biomass accumulation. A comprehensive investigation of plant biodiversity, biomass, and functional traits and subsequent assessment of plant life history strategies in conserved and disturbed forests will aid investigations of regional biodiversity and carbon reserves, contribute data to the TRY and Chinese plant trait databases, and improve ecological management and restoration efforts in east China.

## 1 Introduction

Forests cover approximately 31% of Earth’s land surface and provide important ecosystem services, including biodiversity protection and climate change mitigation (Melillo et al., 1993; Anderson-Teixeira et al., 2015; FAO, 2020). Natural and anthropogenic disturbances, such as climate change, fire, pest outbreak, livestock grazing, and land use change drive environmental heterogeneity and regulate community composition, structure, and function in forests (Zhang and Shangguan, 2006; Turner, 2010; Mori et al., 2018; Wang et al., 2020; Thom and Seidl, 2022; Yang et al., 2022; Patacca et al., 2023; Stritih et al., 2023). Worldwide, forests are increasingly altered by natural and anthropogenic disturbances that occur with variable frequency and/or intensity. Understanding the effects of perturbation and the corresponding strategies that enable plants to grow and reproduce under specific conditions (*e.g.*, habitat type, climate, species composition) in forests under different disturbance histories is important for the development of informed forest management and restoration approaches (MacArthur and Wilson, 1967; Grime, 1977; Meyer et al., 2021; Senf and Seidl, 2021; Edgar and Westfall, 2022; Senf and Seidl, 2022).

Plant biodiversity, biomass, and functional traits in forests are key research areas in disturbance and conservation ecology (Turner, 2010; Newman, 2019; Loto and Bravo, 2020). Biodiversity reflects species composition and diversity and is closely related to community stability and ecosystem function (Petchey and Gaston, 2006; Xu et al., 2016). The intermediate disturbance hypothesis postulates that biodiversity varies with disturbance frequency and/or intensity and usually peaks at intermediate time spans and intensities. Adaptation to disturbance drives significant differences in plant community structure in disturbed forests (Horn, 1975; Connell, 1978; Bongers et al., 2009; Meyer et al., 2021). Biomass is the living organic mass that accumulates as green plants assimilate carbon. It forms the basis of community productivity and carbon storage and can be used as a direct indicator of environmental quality in forest ecosystems (Cannell, 1982). Forest disturbance decreases net primary productivity, resulting in lower biomass and carbon accumulation (Pugh et al., 2019). Plant functional traits refer to a set of plant attributes that may affect the colonization, survival, growth, and death of plants (Violle et al., 2007). Individually or in combination, plant functional traits may indicate an ecosystem’s response to environmental change and can strongly influence ecosystem processes (Cornelissen et al., 2003; Reich et al., 2003). Functional traits are closely linked to disturbances such as fire, biological invasion, and land use change (Verheyen et al., 2003; Pausas et al., 2004; Lamarque et al., 2011). Plant biodiversity, biomass, and functional traits are thus affected by environmental perturbation and may be useful in illuminating the resource acquisition and allocation strategies plants adopt in environments experiencing different types of disturbance.

Deriving from the rise of the Qinghai–Tibet Plateau, east China is covered by a broad area of subtropical forests, especially zonal evergreen broad-leaved forests, which form a unique ecosystem type in the global subtropical area (Editorial Committee of Vegetation Map of the People’s Republic of China (ECVMC), Chinese Academy of Sciences, 2007). This forest has high species diversity and vegetation carbon storage and is important to the regional environment (Song, 2013). However, rapid economic development and population growth, in conjunction with a lack of understanding of the importance of ecological protection, has resulted in multifarious anthropogenic disturbances for the last several decades. Few parcels of primary conserved forests remain and are mostly scattered across a few nature reserves and in remote mountain locations (Fan et al., 2019). Plant species composition and diversity, biomass, and functional trait characteristics differ significantly between anthropogenically disturbed forests and primary conserved forests in this region (Zhu et al., 1997; Li et al., 2018; Huang et al., 2022; Yin, 2022; Luo et al., 2023; Yu et al., 2023). Nevertheless, the influence on biodiversity of specific strategies adopted by plants in response to environmental changes in perturbed and conserved forests have not been reported. Some studies have explored the life history strategies of plants in these types of forests from a functional trait perspective, but these studies only measured leaf treats (Huang et al., 2022; Yin, 2022; Yu et al., 2023).

In this study, we explored the life history strategies of plants from the perspective of associated biodiversity, biomass, and functional traits in a total of 66 east China forests (22 conserved and 44 intermediately disturbed forests) under two different disturbance histories. We evaluated plant species composition, diversity, biomass, and twelve morphological traits (leaf, twig, bark, and stem) for 19 species commonly found in both forest types. Accordingly, the two following predictions were made: (1) Biodiversity, biomass, and functional traits differ significantly between forests with two different disturbance histories. (2) Disturbed forests exhibit higher biodiversity (especially for more shade-intolerant species), with individuals exhibiting higher leaf thickness (LT), bark thickness (BT), leaf tissue density (LTD), leaf dry matter content (LDMC), twig tissue density (TTD) and dry matter content (TDMC), bark tissue density (BTD) and dry matter content (BDMC), and stem tissue density (STD) and dry matter content (SDMC) and lower leaf area (LA) and specific leaf area (SLA), representing a resource conservative strategy with a low growth rate (*i.e.*, low biomass). Conserved forests exhibit lower biodiversity (especially for less shade-intolerant species) and individuals exhibit opposite trait combinations, representing a resource acquisition strategy with a high growth rate (*i.e.*, high biomass). This study will improve understanding of the effects of disturbance on forest community structure and function and provide insights to inform forest conservation practices in the east subtropical zone in China.

## 2 Materials and methods

### 2.1 Study area

Jiulong Mountain straddles the borders of Zhejiang, Fujian, and Jiangxi Provinces in east China and is among the 35 priority areas for biodiversity protection in China (Ministry of Ecology and Environment of the People’s Republic of China (MEEC), 2011). It is located in a mid-subtropical climate zone and has a monsoon climate. The mean annual temperature is 16.2°C, with maximum and minimum temperatures of 42.0°C and −10.5°C, respectively. Mean annual precipitation is 1,856 mm and mean annual sunshine duration is 1,925 h (Zheng et al., 2021). Owing to its remote location and relative inaccessibility, Jiulong Mountain harbors well-conserved native vegetation and a number of endangered and rare animal and plant species, especially in Jiulong Mountain National Nature Reserve (118°49′–118°55′ E, 28°19′–28°24′ N). This site is located in Suichang County, Zhejiang Province and has an area of 55.25 km^2^. It was declared as a Province Reserve in 1983 and was promoted to a National Nature Reserve in 2003. Conservation efforts focus on animals such as *Ursus thibetanus*, *Muntiacus crinifrons* and *Tragopan caboti*, plants such as *Torreya jiulongshanensis*, *Taxus wallichiana* var. *mairei*, and *Bretschneidera sinensis*, and the native vegetation.

### 2.2 Vegetation survey and biomass estimation

Field measurements were collected from the Jiulong Mountain National Nature Reserve and its peripheral protective band. This area has not experienced forest fires for more than 100 years. Anthropogenic disturbances such as forest logging, firewood cutting, forest plantation, and livestock grazing took place both in the reserve (especially in the buffer and experimental zones) and its peripheral protective band before it was established as a Province Reserve in 1983. After it received its protective designation, indigenous people inhabiting the area migrated out of the reserve, and all human activity has been strictly controlled for the last 40 years. However, intermediate anthropogenic disturbance continued to occur in the reserve’s peripheral protective band. *Cunninghamia lanceolata* and *Pinus massoniana* forests and bamboo forests were planted in 1988 and 1993, respectively. Other natural forests were occasionally subjected to disturbances such as firewood cutting and livestock grazing. Following a comprehensive vegetation survey, 22 plots of conserved forest representative of the protected were selected in Jiulong Mountain National Nature Reserve along with 44 disturbed forest plots with vegetation characteristic of the mountain in the peripheral protective band of the reserve. All plots had an area of 20 m × 20 m (**Supplementary Table 1**). Geographical coordinates, elevation, slope, aspect, litter thickness, and outcrop coverage were recorded for each plot. Species identity, diameter at breast height (*D*), and height and crown width were recorded for all woody plants with *D* ≥ 5 cm. The total biomass of each individual woody plant was estimated using biomass allometric models (**Supplementary Table 2**). The biomass of species (most dominant species were included) was estimated using biomass allometric models when available. The biomass of species for whom models were not available was estimated using universal allometric models.

### 2.3 Assessment of plant functional traits

Following the vegetation survey, 19 common species (usually called overlapping species, *Cryptomeria japonica* var. *sinensis*, *C. lanceolata*, *P. massoniana*, *Acer davidii*, *Acer elegantulum*, *Alniphyllum fortunei*, *Castanopsis eyrei*, *Celtis biondii*, *Choerospondias axillaris*, *Cornus hongkongensis* subsp. *elegans*, *Machilus thunbergii*, *Phoebe sheareri*, *Prunus schneideriana*, *Quercus glauca*, *Schima superba*, *Eurya muricata*, *Itea omeiensis*, *Lindera erythrocarpa*, and *Loropetalum chinense*) that were distributed across both conserved and disturbed forests were selected for functional trait evaluation. Ten healthy individuals with similar *D* were selected for each species, with five individuals collected from each forest type. Four branches were collected from each individual, with each one taken from a different position on the sunlit side of the tree canopy using an averruncator. Five mature broad leaves or 10 mature needle leaves and a ~20 cm-long terminal twig were sampled from each branch. 20/40 leaves and four twigs were sampled from each individual. Three bark samples and three stem samples were collected from each individual near *D* using a sickle and an increment borer.

Fresh and dry (oven-dried at 80°C for 72 h) leaf, twig, bark, and stem samples were weighed using an electronic balance (accurate to 0.001 g). LA was determined using a WinFOLIA multipurpose leaf area meter (Regent Instruments, Canada). LT and BT were measured using an electronic vernier caliper (accurate to 0.01 mm). Leaf sample volume was calculated by multiplying LA and LT. Twig, bark, and stem sample volume was measured using the drainage method (Cornelissen et al., 2003). The values of SLA, LTD, LDMC, TTD, TDMC, BTD, BDMC, STD, and SDMC were calculated as shown in **Supplementary Table 3**.

### 2.4 Data analysis

Plant species composition and diversity and biomass were only evaluated for individuals with *D* ≥ 5 cm. Plant diversity was assessed using species richness and the Simpson diversity index. Non-metric multidimensional scaling was used to compare species composition between conserved and disturbed forests. Species were classified as shade-tolerant, shade-intolerant, and neutral (Song, 2013). Principal component analysis (PCA) was used to evaluate the effects of plant species on functional traits and show functional trait distributions among plant species. Trait data were log-transformed before PCA analysis. An independent sample t-test was conducted to determine biomass differences between conserved and disturbed forests and trait differences between plants in conserved and disturbed forests. All statistical analyses were performed using R software version 4.2.0 (R Core Team, 2018).

## 3 Results

**Figure 1:**
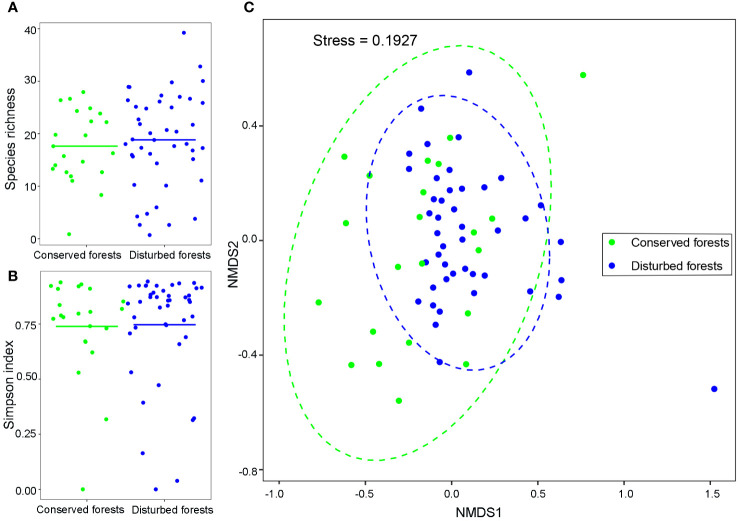
T-test comparisons of species richness **(A)**, Simpson index values **(B)**, and non-metric multidimensional scaling ordination of species composition **(C)** in conserved and disturbed forests in east China. Points correspond to relative species richness **(A)**, Simpson index values **(B)**, and species composition **(C)** in forest plots. Lines indicate mean species richness **(A)** and Simpson index values **(B)**. Species richness and Simpson index values were not significantly different between conserved and disturbed forests (*P* > 0.05).

A total of 7,546 individuals belonging to 195 species were recorded. A combined total of 84 species were identified in conserved and disturbed forests, with 56 species exclusive to conserved forests and 54 species exclusive to disturbed forests. *C. lanceolata*, *S. superba*, *A. fortunei*, *Q. glauca*, and *C. eyrei* were the dominant tree species distributed across both forest types. Only a single individual was identified for 35 species in conserved forests and for 23 species in disturbed forests. The species richness of conserved forests ranged from 1 (pure *C. japonica* forest) to 28 (mixed coniferous broadleaved forest dominated by *C. lanceolata* and *M. thunbergii*), corresponding to an average of 17.64 ± 6.69. The species richness of disturbed forests ranged from 1 (pure *Phyllostachys edulis* forest) to 39 (deciduous broadleaved forest dominated by *A. fortunei* and *C. axillaris*), for an average of 18.84 ± 6.69 (**Figure 1A**). Average Simpson index values were 0.74 ± 0.22 for conserved and 0.75 ± 0.25 for disturbed forests (**Figure 1B**). Disturbed forests had slightly higher species richness and Simpson index values than conserved ones (*P* > 0.05). Species composition was similar for conserved and disturbed forests (**Figure 1C**). Slightly more shade-intolerant (early-succession) species were observed in disturbed forests (44.93%) than in conserved ones (43.57%).

**Figure 2:**
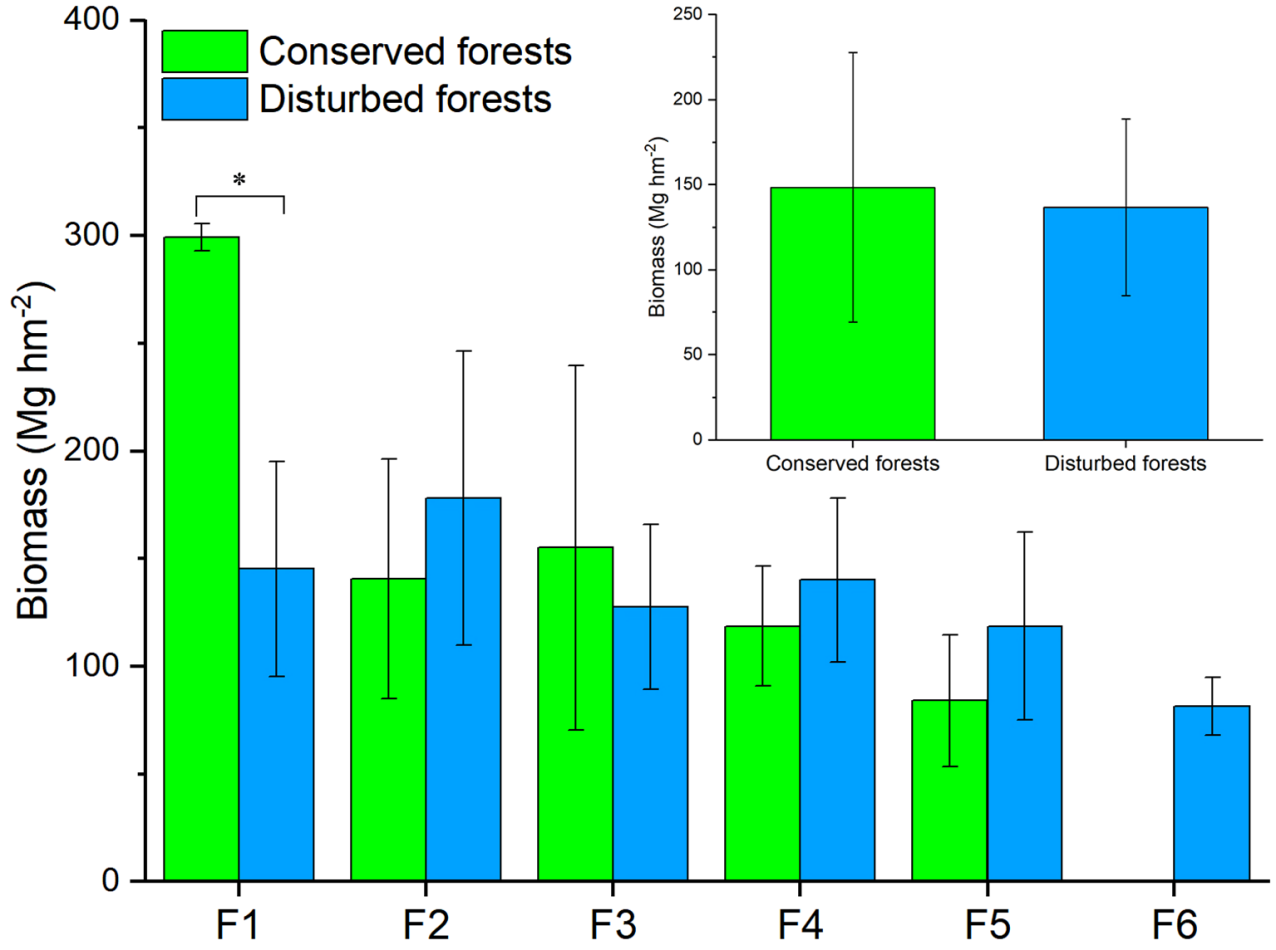
Differences in biomass between conserved and disturbed forests in east China. Significant difference (t-test, *P* < 0.05) is indicated by a star. F1, evergreen broad-leaved forest; F2, evergreen coniferous forest; F3, evergreen and deciduous broad-leaved mixed forest; F4, coniferous and broad-leaved mixed forest; F5, deciduous broad-leaved forest; F6, bamboo forest.

Conserved forest biomass ranged from 53.39 Mg hm^-2^ to 326.42 Mg hm^-2^, averaging 148.53 ± 79.21 Mg hm^-2^. The biomass of disturbed forests ranged from 68.16 Mg hm^-2^ to 268.13 Mg hm^-2^, for an average of 136.74 ± 51.99 Mg hm^-2^. Among conserved forests, high biomass values (> 250 Mg hm^-2^) were recorded for an evergreen and deciduous broadleaved mixed forest dominated by *Liriodendron chinense* and *Quercus multinervis* and for two evergreen broadleaved forests dominated by *Rhododendron simiarum* and *Cyclobalanopsis multinervis*, respectively. Low biomass values (< 70 Mg hm^-2^) were recorded for an evergreen and broadleaved mixed forest dominated by *S. superba* and *Cyclocarya paliurus* and a deciduous broadleaved forest dominated by *Cladrastis wilsonii*. Among disturbed forests, high biomass was recorded for a mature *C. lanceolata* forest and low biomass was recorded for a *Phyllostachys edulis* forest and a *C. lanceolata* forest. T-test results indicated that forests of the same type had similar biomass values (*P* > 0.05, **Figure 2**). For different forest types, conserved evergreen broadleaved forests had significantly higher biomass than disturbed ones (*P* < 0.05), whereas other types of conserved and disturbed forests had similar biomass (*P* > 0.05) (**Figure 2**).

**Figure 3:**
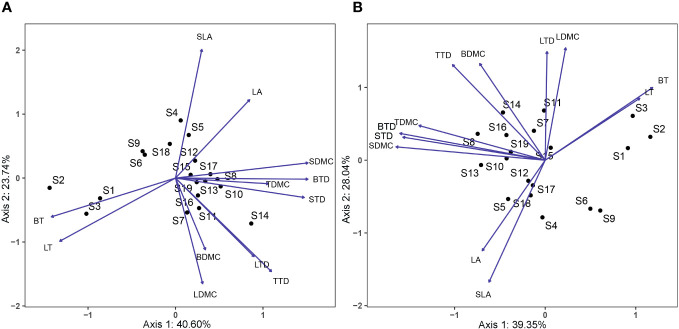
PCA showing the distribution of plant functional traits across common species in conserved **(A)** and disturbed **(B)** forests in east China. Axis1 accounts for 40.60% **(A)** or 39.35% **(B)** of variation, and Axis2 accounts for 23.74% **(A)** or 28.04% **(B)** of variation. LA, leaf area; LT, leaf thickness; SLA, specific leaf area; LTD, leaf tissue density; LDMC, leaf dry matter content; TTD, twig tissue density; TDMC, twig dry matter content; BT, bark thickness, BTD, bark tissue density; BDMC, bark dry matter content; STD, stem tissue density; SDMC, stem dry matter content. S1, *Cryptomeria japonica* var. *Sinensis*; S2, *Cunninghamia lanceolata*; S3, *Pinus massoniana*; S4, *Acer davidii*; S5, *Acer elegantulum*; S6, *Alniphyllum fortunei*; S7, *Castanopsis eyrei*; S8, *Celtis biondii*; S9, *Choerospondias axillaris*; S10, *Cornus hongkongensis* subsp. *elegans*; S11, *Machilus thunbergii*; S12, *Phoebe sheareri*; S13, *Prunus schneideriana*; S14, *Quercus glauca*; S15, *Schima superba*; S16, *Eurya muricata*; S17, *Itea omeiensis*; S18, *Lindera erythrocarpa*; S19, *Loropetalum chinense*.

Functional traits differed between species in conserved and disturbed forests (**Figure 3**). Generally, the 19 dominant overlapping species presented higher LA, SLA, LTD, and BT and lower LT, LDMC, TTD, TDMC, BTD, BDMC, STD, and SDMC in conserved forests than in disturbed forests (**Supplementary Table 4**). LA, SLA, and BT were statistically similar in conserved and disturbed forests (*P* > 0.05), whereas conserved forests had significantly higher LTD and significantly lower LT, LDMC, TTD, TDMC, BTD, BDMC, STD, and SDMC than disturbed ones (*P* < 0.05, **Table 1**). No significant difference was identified for any *P. massoniana* traits between conserved and disturbed forests (**Table 2**). Other species’ traits varied to differing degrees between conserved and disturbed forests (**Table 2**).

**Table 1:** Plant functional traits in conserved and disturbed forests in east China.

Plant functional traits	Conserved forests	Disturbed forests
Leaf area, LA, cm^2^	22.37 ± 15.06 a	19.01 ± 12.27 a
Leaf thickness, LT, mm	0.35 ± 0.15 b	0.46 ± 0.17 a
Specific leaf area, SLA, cm^2^ g	127.12 ± 50.44 a	113.05 ± 47.08 a
Leaf tissue density, LTD, g cm^-3^	0.28 ± 0.09 a	0.23 ± 0.06 b
Leaf dry matter content, LDMC, g g^-1^	0.38 ± 0.05 b	0.43 ± 0.05 a
Twig tissue density, TTD, g cm^-3^	0.48 ± 0.09 b	0.54 ± 0.09 a
Twig dry matter content, TDMC, g g^-1^	0.48 ± 0.05 b	0.53 ± 0.05 a
Bark thickness, BT, mm	0.44 ± 0.30 a	0.40 ± 0.30 a
Bark tissue density, BTD, g cm^-3^	0.47 ± 0.11 b	0.52 ± 0.16 a
Bark dry matter content, BDMC, g g^-1^	0.51 ± 0.07 b	0.55 ± 0.09 a
Stem tissue density, STD, g cm^-3^	0.51 ± 0.11 b	0.56 ± 0.14 a
Stem dry matter content, SDMC, g g^-1^	0.50 ± 0.08 b	0.54 ± 0.11 a

Different letters indicate significant differences between trait values (t-test, *P* < 0.05).

**Table 2:** Differences in plant traits between conserved and disturbed forests in east China.

Plant species/functional traits	Leaf area	Leaf thickness	Specific leaf area	Leaf tissue density	Leaf dry matter content	Twig tissue density	Twig dry matter content	Bark thickness	Bark tissue density	Bark dry matter content	Stem tissue density	Stem dry matter content
*Cryptomeria japonica* var. *Sinensis*	0.315	**0.001**	0.507	**0.008**	0.102	0.199	0.243	0.610	0.550	0.082	0.341	0.953
*Cunninghamia lanceolata*	0.777	0.548	0.085	**0.026**	**0.007**	**0.001**	**0.024**	0.088	**0.039**	0.998	0.945	0.314
*Pinus massoniana*	0.351	0.849	0.165	0.123	0.106	0.313	0.559	0.971	0.240	0.393	0.975	0.711
*Acer davidii*	0.544	0.463	0.114	**0.024**	**0.033**	**0.004**	**0.048**	0.132	0.130	0.071	0.079	0.492
*Acer elegantulum*	0.435	0.179	0.472	0.919	0.202	**0.002**	**0.005**	**0.042**	**0.017**	**0.001**	0.069	0.356
*Alniphyllum fortunei*	0.276	**0.001**	0.934	**0.003**	0.135	0.258	**0.006**	**0.046**	**0.033**	0.083	0.361	0.602
*Castanopsis eyrei*	0.747	0.633	0.936	0.363	**0.001**	**0.021**	0.087	0.225	0.087	0.583	0.260	0.446
*Celtis biondii*	0.550	0.089	**0.045**	0.803	0.097	**0.018**	0.054	0.149	**0.003**	**0.003**	0.155	0.249
*Choerospondias axillaris*	0.491	**0.007**	0.239	0.062	0.152	0.972	0.378	0.247	0.890	0.499	0.641	0.409
*Cornus hongkongensis* subsp. *elegans*	0.596	**0.001**	**0.017**	**0.007**	**0.009**	0.465	0.908	0.073	0.513	0.806	0.150	0.275
*Machilus thunbergii*	0.217	**0.001**	0.183	**0.031**	**0.025**	0.102	**0.002**	**0.006**	0.059	**0.001**	0.227	0.208
*Phoebe sheareri*	0.375	**0.001**	0.274	**0.010**	0.084	0.526	0.208	0.272	0.173	**0.001**	**0.039**	**0.012**
*Prunus schneideriana*	0.097	0.181	0.218	0.091	0.492	**0.014**	0.396	0.114	**0.043**	0.106	**0.016**	**0.015**
*Quercus glauca*	0.235	**0.001**	0.127	**0.001**	**0.004**	0.303	**0.001**	0.196	**0.009**	**0.031**	0.343	**0.038**
*Schima superba*	0.134	**0.001**	**0.001**	**0.001**	**0.008**	**0.001**	**0.001**	**0.027**	**0.001**	**0.001**	0.070	0.085
*Eurya muricata*	**0.010**	**0.001**	0.220	**0.030**	**0.004**	0.370	**0.001**	**0.002**	0.234	**0.006**	**0.001**	**0.002**
*Itea omeiensis*	0.063	**0.001**	0.889	**0.001**	0.444	0.784	0.108	0.100	0.993	0.897	0.841	0.184
*Lindera erythrocarpa*	0.671	**0.001**	0.627	**0.044**	**0.047**	**0.049**	**0.007**	0.765	**0.032**	**0.001**	**0.021**	**0.005**
*Loropetalum chinense*	0.339	**0.026**	0.399	**0.048**	**0.008**	**0.010**	**0.020**	0.696	**0.021**	**0.001**	0.076	**0.030**

Significant differences (*P* < 0.05) are highlighted in bold.

## 4 Discussion

Vegetation–disturbance interactions have long been the focus of intense research in disturbance and conservation ecology. A single index, such as community biodiversity, is often used to evaluate the effects of natural and anthropogenic disturbances on vegetation (Bongers et al., 2009; Gosper et al., 2013; Meyer et al., 2021), but biodiversity-only approaches do not allow the complete characterization of vegetation–disturbance interactions (Mouillot et al., 2013). This study assessed plant biodiversity, biomass, and functional traits in subtropical forests with two different disturbance histories, and the results aim to improve understanding of vegetation–disturbance interactions. However, this study has some shortcomings. Though our exclusion of individuals with *D <* 5 *cm* did not have a considerable effect on biomass estimates, it may have had a sizeable effect on our estimates of species composition and diversity. Future studies should consider herbaceous vegetation and woody individuals with *D* < 5 cm. Moreover, inter- and intraspecific variation in some plant functional traits, such as BT, LA, LT, and SLA, was high, and a sample size of five individuals may not have been sufficiently large.

Global vegetation distributions and plant community composition, structure, and function are determined by climate but are also strongly influenced by other environmental factors at the regional and local scales, including land use change, disturbance, topography, and soil (Zhang, 1993; Thuiller et al., 2004; Alessa and Chapin, 2008; Gillman et al., 2015; Liu and Ma, 2015; Liu et al., 2016). In this study, conserved and disturbed forests were adjacent to one another and shared similar climates and local habitats but had different disturbance histories. Our assessment of life history strategy differences based on plant biodiversity, biomass, and functional traits is robust because of the direct exclusion of the effects of other environmental factors. Assessing plant functional traits using overlapping plant species also allowed us to exclude the effect of species identity (Liu et al., 2023).

The intermediate disturbance hypothesis suggests that biodiversity peaks at intermediate time spans and intensities (Horn, 1975; Connell, 1978; Meyer et al., 2021). In this study, slightly higher biodiversity was observed in intermediately disturbed forests than in conserved forests. This is because only plant individuals with *D* ≥ 5 cm were sampled, and understory plants are more strongly influenced by disturbance (Meyer et al., 2021; Huang et al., 2022). The biomass of subtropical forests disturbed by anthropogenic fire, forest logging, and livestock grazing is lower than that of primary zonal evergreen broad-leaved forest in southern China (Li et al., 2018; Luo et al., 2023). This study also found that the disturbed evergreen broad-leaved forests had significantly lower biomass relative to primary conserved forests. Due to its excellent protection regime, conserved forests in the Jiulong Mountain National Nature Reserve have higher biomass than forests in east China (Dai et al., 2017). Plant scientists have focused more attention on the functional traits of natural vegetation than on functional traits in artificial and anthropogenically disturbed vegetation (Liu and Ma, 2015), with most of the data in global and Chinese plant trait databases collected from natural vegetation (Kattge et al., 2011; Kattge et al., 2020; Wang et al., 2018). Here and in the other few studies conducted in Chinese subtropical forests, disturbed forests had significantly higher LDMC, lower LA and SLA, and similar LTD compared with conserved forests (Huang et al., 2022).

Biodiversity, biomass, functional traits, and their interactions can reveal responses and adaptation to specific environmental conditions, resource acquisition and utilization strategies, and the corresponding life history strategies that adopted by vegetation to adapt to various environments (Díaz et al., 2016; Pugh et al., 2019; Meyer et al., 2021). Plants employing a resource acquisition strategy usually grow rapidly (high photosynthetic rate), with high leaf turnover, N content, and SLA, while those adopting a resource conservative strategy usually grow slowly (low photosynthetic rate) with relatively slow leaf turnover, N content, and SLA (Chen and Xu, 2014). Plants tend to adopt different life history strategies when they grow in different environments (Liu et al., 2023). Such environmentally-mediated differences in species composition and life history strategies could reduce niche overlap, maintain high biodiversity, and strengthen ecosystem stability (Liu and Ma, 2015). In this study, we found that individuals in disturbed forests allocate more resources to cope with unfavorable conditions than to grow. As such, they adopt a resource conservation strategy, increasing biodiversity and LT, LDMC, TTD, TDMC, BTD, BDMC, STD, and SDMC while reducing SLA to conserve resources and grow slower (lower biomass). In contrast, conserved forests allocate more resources for rapid growth (higher biomass), adopting a resource acquisition strategy and reducing biodiversity.

Anthropogenic disturbance has impacted forests in east China for much of recorded history, and land cover is dominated by disturbed forests and degraded vegetation such as shrub and grass communities. Remaining primary evergreen broad-leaved forest covers only 4% of its total distribution area in east China (Song, 2013; Liu et al., 2023). Ecosystem services, such as water conservation, biodiversity protection, and carbon sequestration could be enhanced by restoring climax evergreen broad-leaved forests in these degraded areas, mitigating the effects of regional and global environmental changes. A comprehensive comparison of plant biodiversity, biomass, and functional traits and subsequent assessment of life history strategy differences between conserved and disturbed forests will provide basic data for regional biodiversity and carbon inventories and for global and Chiense plant trait databases. It will also guide approaches to ecological management and restoration of degraded evergreen broad-leaved forest in east China.

## 5 Conclusion

Overall, our assessment of plant biodiversity, biomass, and functional traits suggests that individuals in disturbed forests adopt a resource conservation strategy, enhance biodiversity, and develop multiple functional traits such as high LT, LDMC, TTD, TDMC, BTD, BDMC, STD, and SDMC and low SLA to reduce the rate of biomass accumulation. Vegetation in conserved forests adopts a resource acquisition strategy, reduces biodiversity, and develops converse trait combinations to support high rates of biomass accumulation. A better understanding of the life history strategies of disturbed forests will improve forest conservation and restoration efforts in east China.

## Data availability statement

The original contributions presented in the study are included in the article/**Supplementary Material**. Further inquiries can be directed to the corresponding authors.

## Author contributions

WZ and LL conceived and designed the research. JL, HX, ZZ, YW, JC, and LL contributed to the field work. JL, HX, and LL analyzed the data and wrote the first draft with substantial input from WZ, JN, and MY. All authors contributed to the article and approved the submitted version.
