# Supplementary material for: Plant life history strategies vary in subtropical forests with different disturbance histories: an assessment of biodiversity, biomass, and functional traits
Source: Front Plant Sci. 2024 Jan 10;14:1230149. doi: 10.3389/fpls.2023.1230149 (PMC10806164; doi:10.3389/fpls.2023.1230149)
Supplement: Supplementary file 1 [file Table_1.docx]

Supplementary Material

**Supplementary Table 1.** Basic information for 22 conserved forest plots and 44 disturbed forest plots in Zhejiang Province, east China. D, H and N, average diameter and height, and individual number of woody plants with diameter at breast height (D) ≥ 5 cm.

| Disturbance history | Number | Location | Elevation (m) | Forest type | Dominant species | Species richness | Simpson index | Biomass (Mg hm^-2^) | D (cm) | H (m) | N |
| --- | --- | --- | --- | --- | --- | --- | --- | --- | --- | --- | --- |
| Conserved forest | 1 | 118°52′34.52′′ E  28°20′16.42′′ N | 1340 | Evergreen coniferous forest | *Pinus taiwanensis* | 13 | 0.77 | 140.51 | 10.01 | 8.00 | 164 |
|  | 2 | 118°52′16.70′′ E  28°20′17.04′′ N | 1375 | Evergreen coniferous forest | *Cryptomeria japonica* var. *sinensis* | 1 | 0.00 | 246.27 | 21.84 | 20.51 | 53 |
|  | 3 | 118°52′31.48′′ E  28°21′13.74′′ N | 1582 | Evergreen broad-leaved forest | *Rhododendron simiarum*, *Schima superba* | 11 | 0.32 | 305.55 | 12.20 | 7.40 | 261 |
|  | 4 | 118°52′41.40′′ E  28°21′20.67′′ N | 1649 | Coniferous and broad-leaved mixed forest | *Pinus taiwanensis*; *Quercus multinervis* | 22 | 0.84 | 171.57 | 12.44 | 8.11 | 113 |
|  | 5 | 118°52′49.71″ E  28°21′20.53″ N | 1643 | Evergreen coniferous forest | *Pinus taiwanensis* | 8 | 0.62 | 88.39 | 14.29 | 9.17 | 79 |
|  | 6 | 118°52′45.23″ E  28°21′6.29″ N | 1654 | Evergreen broad-leaved forest | *Quercus multinervis* | 14 | 0.67 | 292.99 | 13.20 | 9.90 | 111 |
|  | 7 | 118°52′34.83″ E  28°20′45.65″ N | 1579 | Evergreen and deciduous broad-leaved mixed forest | *Schima superba*, *Symplocos coreana* | 13 | 0.67 | 99.29 | 7.98 | 6.32 | 141 |
|  | 8 | 118°52′34.69″ E  28°20′39.68″ N | 1497 | Coniferous and broad-leaved mixed forest | *Cunninghamia lanceolata*, *Daphniphyllum macropodum*, *Pinus taiwanensis* | 20 | 0.80 | 100.86 | 7.26 | 6.47 | 169 |
|  | 9 | 118°52′9.69′′ E  28°21′11.43′′ N | 1250 | Evergreen and deciduous broad-leaved mixed forest | *Lithocarpus brevicaudatus*, *Cladrastis wilsonii* | 26 | 0.92 | 217.42 | 12.66 | 10.79 | 90 |
|  | 10 | 118°51′11.46′′ E  28°22′2.36′′ N | 687 | Evergreen and deciduous broad-leaved mixed forest | *Cyclocarya paliurus*, *Phoebe sheareri* | 16 | 0.85 | 164.64 | 13.00 | 7.51 | 84 |
|  | 11 | 118°51′11.04′′ E  28°22′10.51′′ N | 690 | Deciduous broad-leaved forest | *Choerospondias axillaris*, *Cyclocarya paliurus*, *Alniphyllum fortunei*, *Meliosma myriantha* var. *discolor* | 25 | 0.93 | 114.67 | 11.85 | 8.09 | 76 |
|  | 12 | 118°53′28.29′′ E  28°22′43.51′′ N | 649 | Evergreen and deciduous broad-leaved mixed forest | *Choerospondias axillaris*, *Phoebe sheareri* | 20 | 0.74 | 123.14 | 11.71 | 8.38 | 91 |
|  | 13 | 118°50′44.85″ E  28°23′39.5″ N | 507 | Evergreen coniferous forest | *Pinus massoniana* | 12 | 0.82 | 121.48 | 14.72 | 9.05 | 45 |
|  | 14 | 118°53′29.33″ E  28°21′48.77″ N | 969 | Evergreen and deciduous broad-leaved mixed forest | *Schima superba*, *Cyclocarya paliurus* | 24 | 0.94 | 61.70 | 8.23 | 6.24 | 76 |
|  | 15 | 118°53′34.35″ E  28°21′48.53″ N | 1000 | Deciduous broadleaved forest | *Cladrastis wilsonii*, *Acer davidii* | 13 | 0.80 | 53.39 | 10.37 | 7.41 | 51 |
|  | 16 | 118°50′57.55″ E  28°22′31.33″ N | 716 | Evergreen and deciduous broad-leaved mixed forest | *Machilus thunbergii*, *Choerospondias axillaris* | 27 | 0.91 | 93.37 | 9.68 | 6.59 | 77 |
|  | 17 | 118°51′0.30″ E  28°22′30.19″ N | 676 | Coniferous and broad-leaved mixed forest | *Cunninghamia lanceolata*, *Machilus thunbergii* | 28 | 0.92 | 108.95 | 10.78 | 9.01 | 77 |
|  | 18 | 118°50′52.88′′ E  28°23′11.29′′ N | 540 | Coniferous and broad-leaved mixed forest | *Cunninghamia lanceolata*, *Adinandra millettii* | 22 | 0.79 | 94.81 | 7.95 | 5.43 | 188 |
|  | 19 | 118°50′57.01′′ E  28°23′11.42′′ N | 633 | Evergreen coniferous forest | *Cunninghamia lanceolata* | 18 | 0.53 | 106.37 | 9.01 | 7.41 | 138 |
|  | 20 | 118°53′26.63′′ E  28°22′52.82′′ N | 747 | Coniferous and broad-leaved mixed forest | *Schima superba*, *Cunninghamia lanceolata* | 15 | 0.73 | 138.57 | 11.13 | 6.96 | 101 |
|  | 21 | 118°53′27.74′′ E  28°22′49.33′′ N | 636 | Coniferous and broad-leaved mixed forest | *Cunninghamia lanceolata*, *Schima superba* | 24 | 0.78 | 97.20 | 9.63 | 6.52 | 101 |
|  | 22 | 118°51′59.71′′ E  28°20′50.12′′ N | 1388 | Evergreen and deciduous broad-leaved mixed forest | *Liriodendron chinense*, *Quercus multinervis* | 16 | 0.91 | 326.42 | 17.55 | 8.23 | 41 |
| Disturbed forest | 1 | 118°45′57.21″ E  28°17′7.06″ N | 673 | Evergreen broad-leaved forest | *Quercus glauca*, *Lithocarpus hancei* | 17 | 0.86 | 89.26 | 8.92 | 9.68 | 92 |
|  | 2 | 118°46′1.74″ E  28°16′52.67″ N | 698 | Evergreen broad-leaved forest | *Daphniphyllum oldhamii* | 25 | 0.92 | 102.39 | 10.44 | 8.98 | 80 |
|  | 3 | 118°46′11.43″ E  28°16′34.84″ N | 738 | Coniferous and broad-leaved mixed forest | *Cunninghamia lanceolata*, *Pinus massoniana*, *Quercus glauca*, *Daphniphyllum oldhamii* | 15 | 0.86 | 176.84 | 14.79 | 12.03 | 58 |
|  | 4 | 118°48′30.00″ E  28°20′29.00″ N | 572 | Evergreen coniferous forest | *Pinus massoniana*, *Cunninghamia lanceolata* | 6 | 0.66 | 217.33 | 17.68 | 11.69 | 57 |
|  | 5 | 118°48′45.23″ E  28°20′9.63″ N | 595 | Bamboo forest | *Phyllostachys edulis* | 4 | 0.04 | 69.72 | 10.38 | 12.05 | 154 |
|  | 6 | 118°48′51.29″ E  28°19′17.91″ N | 667 | Coniferous and broad-leaved mixed forest | *Cunninghamia lanceolata*, *Alniphyllum fortunei* | 11 | 0.78 | 136.30 | 13.04 | 9.07 | 76 |
|  | 7 | 118°49′17.00″ E  28°19′22.00″ N | 662 | Coniferous and broad-leaved mixed forest | *Alniphyllum fortunei*, *Cunninghamia lanceolata* | 14 | 0.74 | 131.45 | 10.57 | 8.77 | 108 |
|  | 8 | 118°49′24.00″ E  28°19′27.00″ N | 697 | Coniferous and broad-leaved mixed forest | *Phyllostachys edulis*, *Cunninghamia lanceolata* | 3 | 0.39 | 121.75 | 11.73 | 11.46 | 149 |
|  | 9 | 118°50′10.00″ E  28°19′32.00″ N | 845 | Deciduous broad-leaved forest | *Prunus schneideriana* | 10 | 0.77 | 103.53 | 13.57 | 8.22 | 68 |
|  | 10 | 118°49′54.59″ E  28°19′22.84″ N | 771 | Evergreen coniferous forest | *Cunninghamia lanceolata* | 10 | 0.53 | 268.13 | 12.52 | 9.86 | 124 |
|  | 11 | 118°48′55.79″ E  28°18′52.11″ N | 750 | Deciduous broad-leaved forest | *Choerospondias axillaris*, *Acer davidii* | 25 | 0.92 | 81.61 | 11.19 | 8.04 | 55 |
|  | 12 | 118°48′50.56″ E  28°18′30.40″ N | 780 | Evergreen and deciduous broad-leaved mixed forest | *Machilus leptophylla*, *Liquidambar formosana* | 17 | 0.88 | 201.44 | 15.09 | 8.75 | 55 |
|  | 13 | 118°46′58.93″ E  28°17′7.18″ N | 748 | Evergreen broad-leaved forest | *Schima superba*, *Quercus glauca* | 20 | 0.89 | 196.89 | 11.67 | 8.72 | 110 |
|  | 14 | 118°47′1.38″ E  28°16′53.41″ N | 720 | Coniferous and broad-leaved mixed forest | *Alniphyllum fortunei*, *Cunninghamia lanceolata* | 26 | 0.88 | 180.30 | 11.24 | 9.45 | 121 |
|  | 15 | 118°47′4.00″ E  28°17′30.00″ N | 955 | Evergreen and deciduous broad-leaved mixed forest | *Schima superba*, *Platycarya strobilacea*, *Castanopsis eyrei* | 21 | 0.88 | 105.80 | 9.36 | 8.16 | 139 |
|  | 16 | 118°47′13.80″ E  28°17′34.77″ N | 1080 | Evergreen and deciduous broad-leaved mixed forest | *Schima superba*, *Betula luminifera*, *Choerospondias axillaris* | 16 | 0.71 | 177.84 | 10.09 | 8.48 | 190 |
|  | 17 | 118°46′46.79″ E  28°17′28.56″ N | 647 | Evergreen and deciduous broad-leaved mixed forest | *Machilus thunbergii*, *Choerospondias axillaris* | 33 | 0.93 | 100.46 | 8.88 | 7.07 | 114 |
|  | 18 | 118°47′21.07″ E  28°17′5.46″ N | 688 | Deciduous broad-leaved forest | *Alniphyllum fortunei*, *Lindera erythrocarpa* | 22 | 0.82 | 92.20 | 9.29 | 8.66 | 113 |
|  | 19 | 118°46′50.00″ E  28°17′13.00″ N | 675 | Coniferous and broad-leaved mixed forest | *Cunninghamia lanceolata*, *Alniphyllum fortunei*, *Morella rubra* | 23 | 0.73 | 135.40 | 9.97 | 7.23 | 157 |
|  | 20 | 118°47′16.79″ E  28°17′11.64″ N | 726 | Evergreen and deciduous broad-leaved mixed forest | *Choerospondias axillaris*, *Machilus thunbergii* | 30 | 0.92 | 128.49 | 11.69 | 7.44 | 82 |
|  | 21 | 118°47′31.76″ E  28°17′2.94″ N | 745 | Deciduous broad-leaved forest | *Alniphyllum fortunei*, *Acer davidii* | 20 | 0.86 | 106.54 | 10.10 | 7.68 | 99 |
|  | 22 | 118°45′27.04″ E  28°17′45.03″ N | 611 | Evergreen coniferous forest | *Cunninghamia lanceolata* | 26 | 0.78 | 108.26 | 9.25 | 8.22 | 115 |
|  | 23 | 118°45′10.13″ E  28°17′40.00″ N | 690 | Evergreen and deciduous broad-leaved mixed forest | *Machilus leptophylla*, *Machilus thunbergii*, *Alniphyllum fortunei* | 27 | 0.94 | 83.14 | 9.46 | 8.28 | 97 |
|  | 24 | 118°45′29.26″ E  28°17′38.50″ N | 662 | Coniferous and broad-leaved mixed forest | *Cunninghamia lanceolata*, *Cryptomeria japonica* var. *Sinensis*, *Photinia schneideriana* | 29 | 0.94 | 68.16 | 9.05 | 7.80 | 82 |
|  | 25 | 118°45′24.43″ E  28°17′30.99″ N | 742 | Evergreen and deciduous broad-leaved mixed forest | *Machilus thunbergii*, *Alniphyllum fortunei* | 27 | 0.90 | 111.54 | 9.28 | 8.46 | 125 |
|  | 26 | 118°45′41.48″ E  28°17′56.81″ N | 636 | Evergreen coniferous forest | *Cunninghamia lanceolata* | 18 | 0.47 | 159.52 | 10.88 | 7.94 | 139 |
|  | 27 | 118°45′20.53″ E  28°17′49.08″ N | 692 | Evergreen broad-leaved forest | *Castanopsis eyrei*, *Daphniphyllum oldhamiil*, *Quercus glauca*, *Lithocarpus glaber* | 26 | 0.92 | 109.40 | 8.32 | 8.10 | 151 |
|  | 28 | 118°45′3.51″ E  28°17′32.80″ N | 780 | Deciduous broad-leaved forest | *Alniphyllum fortunei*, *Liquidambar formosana* | 19 | 0.87 | 120.92 | 9.86 | 9.00 | 108 |
|  | 29 | 118°47′5.52″ E  28°18′55.10″ N | 704 | Deciduous broad-leaved forest | *Alniphyllum fortunei*, *Choerospondias axillaris* | 18 | 0.87 | 103.79 | 9.94 | 9.24 | 94 |
|  | 30 | 118°47′6.40″ E  28°18′35.80″ N | 708 | Deciduous broad-leaved forest | *Alniphyllum fortunei*, *Choerospondias axillaris* | 39 | 0.92 | 221.69 | 9.75 | 8.92 | 211 |
|  | 31 | 118°45′33.10″ E  28°17′47.00″ N | 741 | Evergreen broad-leaved forest | *Lithocarpus hancei* | 30 | 0.93 | 108.15 | 9.69 | 7.52 | 112 |
|  | 32 | 118°45′1.00″ E  28°17′31.00″ | 758 | Evergreen and deciduous broad-leaved mixed forest | *Phoebe shearer*i, *Machilus leptophylla*, *Alniphyllum fortunei* | 19 | 0.75 | 111.42 | 9.20 | 6.91 | 123 |
|  | 33 | 118°46′7.23″ E  28°16′29.66″ N | 759 | Coniferous and broad-leaved mixed forest | *Cunninghamia lanceolata*, *Schima superba*, *Pinus massoniana* | 22 | 0.85 | 109.52 | 11.58 | 9.73 | 117 |
|  | 34 | 118°46′7.08″ E  28°16′20.18″ N | 807 | Evergreen broad-leaved forest | *Castanopsis eyrei*, *Schima superba* | 16 | 0.69 | 98.95 | 9.88 | 7.25 | 124 |
|  | 35 | 118°46′7.00″ E  28°16′38.17″ N | 500 | Evergreen broad-leaved forest | *Castanopsis eyrei* | 29 | 0.87 | 129.07 | 9.00 | 8.98 | 155 |
|  | 36 | 118°46′8.62″ E  28°16′26.04″ N | 813 | Coniferous and broad-leaved mixed forest | *Pinus massoniana*, *Cunninghamia lanceolata*, *Schima superba* | 16 | 0.85 | 209.41 | 13.45 | 9.33 | 104 |
|  | 37 | 118°45′52.83″ E  28°16′59.13″ N | 744 | Evergreen broad-leaved forest | *Quercus stewardiana*, *Schima superba* | 27 | 0.91 | 176.81 | 8.60 | 8.50 | 198 |
|  | 38 | 118°46′2.48″ E  28°17′13.63″ N | 758 | Evergreen broad-leaved forest | *Castanopsis eyrei*; *Daphniphyllum oldhamii* | 17 | 0.83 | 214.14 | 11.18 | 8.70 | 133 |
|  | 39 | 118°47′21.03″ E  28°19′1.28.00″ N | 784 | Coniferous and broad-leaved mixed forest | *Cunninghamia lanceolata*, *Cryptomeria japonica* var. *sinensis*, *Schima superba*, *Machilus thunbergii* | 25 | 0.91 | 132.01 | 10.12 | 8.89 | 119 |
|  | 40 | 118°47′0.08″ E  28°18′45.81″ N | 733 | Evergreen broad-leaved forest | *Quercus phillyreoides*, *Distylium myricoides* | 18 | 0.84 | 226.50 | 9.99 | 6.34 | 159 |
|  | 41 | 118°47′48.04″ E  28°20′54.38″ N | 538 | Bamboo forest | *Phyllostachys edulis* | 5 | 0.16 | 100.31 | 10.33 | 11.96 | 183 |
|  | 42 | 118°49′37.79″ E  28°19′38.64″ N | 702 | Evergreen coniferous forest | *Pinus massoniana* | 4 | 0.32 | 79.45 | 10.20 | 5.33 | 80 |
|  | 43 | 118°48′19.4″ E  28°20′33.41″ N | 558 | Bamboo forest | *Phyllostachys edulis* | 1 | 0.00 | 74.18 | 10.72 | 11.81 | 158 |
|  | 44 | 118°47′47.53″ E  28°21′0.71″ N | 564 | Evergreen coniferous forest | *Cunninghamia lanceolata*, *Pinus massoniana* | 3 | 0.31 | 236.35 | 14.07 | 9.92 | 104 |

**Supplementary Table 2** Biomass allometric models for subtropical forests in Zhejiang Province, east China. Units for biomass, diameter at breast height (D), and height (H) are kg, cm and m, respectively. W_t_, total biomass; W_a_, aboveground biomass; W_u_, underground biomass; W_l_, leaf biomass; W_b_, branch biomass; W_s_, stem biomass.

| Species | Biomass allometric models | Correlation coefficients | References |
| --- | --- | --- | --- |
| *Cunninghamia lanceolata* | W_t_ = 0.0603(D^2^H)^0.9312^ (D≥9.0)  W_t_ = 1.1687 + 0.2375D^2^ (D<9.0) | R^2^ = 0.958  R = 0.972 | Huang et al., 2011  Hui et al., 1988 |
| *Phyllostachys edulis* | W_t_ = 0.105D^2.176^ | R^2^ = 0.697 | Lu et al., 2018 |
| *Eurya muricata* | W_a_ = 0.7059 × 0.313375 × πD^2^  W_u_ = (35.070(D^2^H)^0.6499^)/1000 | R^2^ = 0.939  R = 0.997 | Ali et al., 2014  Chen et al., 1993 |
| *Pinus taiwanensis* | W_l_ = 0.026D^2.168^  W_b_ = 0.015D^2.047^  W_s_ = 2.471 + 0.305(D^2^H)^0.528^  W_u_ = 0.047D^1.037^ | R^2^ = 0.952  R^2^ = 0.963  R^2^ = 0954  R^2^ = 0.948 | Sun et al., 2022 |
| *Rhododendron latoucheae* | W_a_ = 0.2212D^1.9932^  W_u_ = (35.070(D^2^H)^0.6499^)/1000 | R^2^ = 0.920  R = 0.997 | Lin et al., 2012  Chen et al., 1993 |
| *Schima superba* | W_t_ = 0.0308(D^2^H)^1.0118^ | R^2^ = 0.990 | Luo et al., 2016 |
| *Pinus massoniana* | W_t_ = 0.191D^2.197^ | R = 0.948 | Xu et al., 2011 |
| *Rhododendron ovatum* | W_t_ = 0.289D^1.914^ | R^2^ = 0.951 | Xia et al., 2014 |
| *Phoebe sheareri* | W_t_ = -55.7397 + 14.7759D | R = 0.928 | Chen et al., 1989 |
| *Cryptomeria japonica* var. *sinensis* | W_t_ = 0.2655(D^2^H)^0.7076^ | R = 0.993 | Yao et al, 1997 |
| *Alniphyllum fortunei* | W_t_ = 0.0514(D^2^H)^0.9393^ | R^2^ = 0.940 | Zuo et al., 2015 |
| *Machilus thunbergii* | W_a_ = e ^(–3.51 + 2.59 × Ln D)^  W_u_ = (35.070(D^2^H)^0.6499^)/1000 | R^2^ = 0.990  R = 0.997 | Ali et al., 2015  Chen et al., 1993 |
| *Castanopsis eyrei* | W_t_ = 0.030379216(D^2^H)^1.023314481^ | R^2^ = 0.994 | Ding, 2014 |
| *Loropetalum chinense* | W_a_ = 0.1599D^2.35119^  W_u_ = (35.070(D^2^H)^0.6499^)/1000 | R^2^ = 0.990  R = 0.997 | Lin et al., 2012  Chen et al., 1993 |
| *Choerospondias axillaris* | W_t_ = (86.589(D^2^H)^0.8699^)/1000 (D≥16.3)  W_t_ = 0.0932(D^2^H)^0.8837^ (D<16.3) | R = 0.988  R^2^=0.990 | Chen et al., 1993  Luo et al., 2016 |
| *Quercus glauca*, *Quercus stewardiana*, *Quercus sessilifolia*, *Quercus multinervis* | W_t_= -64.0020 + 12.8340D (D≥5.5)  W_t_ = 1.1963D^1.4829^ (D<5.5) | R^2^ = 0.978  R^2^ = 0.983 | Yu., 1999  Zheng., 2008 |
| *Dalbergia hupeana* | W_t_ = (86.589 (D^2^H)^0.8699^ )/1000 (D≥10)  W_t_ = 0.051(D^2^H)^1.032^ (D<10) | R = 0.988  R = 0.970 | Chen et al., 1993  Zhang., 2016 |
| *Lithocarpus glaber*, *Lithocarpus brevicaudatus* | W_t_ = 0.4086D^2.0880^ | R^2^ = 0.930 | Zuo et al., 2015 |
| *Daphniphyllum oldhamii* | W_t_ = 0.1726D^2.3686^ | R^2^ = 0.960 | Zuo et al., 2015 |
| *Liquidambar formosana* | W_t_ = 0.0761(D^2^H)^0.9078^ | R^2^ = 0.980 | Luo et al., 2016 |
| *Platycarya strobilacea* | W_a_ = 0.0174(D^2^H)+21.767  W_u_ = (38.031 (D^2^H)^0.5902^)/1000 | R^2^ = 0.862  R = 0.993 | Liu et al., 2020  Chen et al., 1993 |
| *Morella rubra* | W_t_ = (136.175(D^2^H)^0.7897^)/1000 (H≥3.9)  W_t_ = 0.6454H^3.1558^  (H<3.9) | R = 0.999  R = 0.616 | Chen et al., 1993  Penf et al., 2022 |
| *Quercus phillyreoides* | W_t_ = 0.174D^2.39^ | R^2^ = 0.947 | Zhang et al., 2015 |
| *Cyclobalanopsis myrsinifolia* | W_t_ = 0.0617(D^2^H) + 2.9528 | R = 0.999 | Yang et al., 2015 |
| *Symplocos sumuntia* | W_a_ = 0.7797 × 0.2062 × πD^2^  W_u_ = (35.070(D^2^H)^0.6499^)/1000 | R^2^ = 0.942  R = 0.997 | Ali et al., 2014  Chen et al., 1993 |
| *Vaccinium mandarinorum* | W_a_ = 0.7 × 0.29192 × πD^2^  W_u_ = (35.070(D^2^H)^0.6499^)/1000 | R^2^ = 0.978  R = 0.997 | Ali et al., 2014  Chen et al., 1993 |
| *Syzygium buxifolium* | W_a_ = 0.7 × (0.04 + 0.28075 × πD^2^)  W_u_ = (35.070(D^2^H)^0.6499^)/1000 | R^2^ = 0.947  R = 0.997 | Ali et al., 2014  Chen et al., 1993 |
| *Adinandra millettii* | W_a_= e×(-3.83 +1.99 × Ln D + 0.860 × Ln H)  W_u_ = (35.070(D^2^H)^0.6499^)/1000 | R^2^ = 0990  R = 0.997 | Ali et al., 2015  Chen et al., 1993 |
| *Diospyros japonica* | W_a_ = e×(-5.57 + 1.89 × Ln D + 1.16 × Ln H + 2.72 × 0.53)  W_u_ = (38.031 (D^2^H)^0.5902^)/1000 | R^2^ = 0.99  R = 0.993 | Ali et al., 2015  Chen et al., 1993 |
| *Camellia cuspidata* | W_a_= 0.7234 × 0.30405 × πD^2^  W_u_ = (35.070(D^2^H)^0.6499^)/1000 | R^2^ = 0.914  R = 0.997 | Ali et al., 2014  Chen et al., 1993 |
| Other evergreen species | W_t_ = (136.175(D^2^H)^0.7897^)/1000 | R = 0.999 | Chen et al., 1993 |
| Other deciduous species | W_t_ = (86.589(D^2^H)^0.8699^)/1000 | R = 0.988 | Chen et al., 1993 |

References:

Ali, A., Ma, W. J., Yang, X, D., Sun, B. W., and Xu, M. S. (2014). Biomass and carbon stocks in *schima superba* dominated subtropical science. *Journal of forest science* 60, 198–207.

Ali, A., Xu, M. S., Zhao, Y. T., Zhang, Q. Q., Zhou, L. L., Yang, X. D., et al. (2015). Allometric biomass equations for shrub and small tree species in subtropical China. *Silva Fennica* 49, 1–10.

Chen, H., Ren, C. H., and Zhen, L. P. (1989). Study of biomass for the *Phoebe bournei* stands. *Journal of Fujian College of forestry*. 9, 411–417.

Chen, Q. C , and Shen, Q. (1993). Studies on the biomass models of the tree stratum of secondary *Gycloblanopsis glauca* forest in Zhejiang. *Acta Phytoecologica et Geobotanica Sinica* 17, 38–47.

Ding, Z. F. (2014). Biomass and growth models of four major tree species in subtropical evergreen broad-leaved forest in Anhui. *Journal of Anhui Agricultural University* 41, 859–865.

Huang, X. S., Wu, C. Z., Hong, W., Li, Z. K., and Cheng, Z. P. (2011). The relationship between stand density and biomass of two rotation Chinese fir plantations. *Journal of Fujian College of Forestry* 31, 102–105.

Hui, G. Y., Tong, S. Z., Liu, J. F., and Luo, Y. W. (1988). Study on the afforestation density of *Cunninghamia Lanceolata* I*.* The effect of density on biomass of Young Growth of *C. Lanceolata*. *Forest Research* 1, 413–417.

Lin, D. M., Lai, J. S., Muller-Landau, H. C., Mi, X. C., and Ma, K. P. (2012). Topographic variation in aboveground biomass in a subtropical evergreen broad-leaved forest in China. *PLoS One* 7, E48244.

Liu, L. B., Zhou, Y. C., Cheng, A. Y., Wang, S. J., Cai, X. L., and Ni, J. (2020). Aboveground biomass estimate of a karst forest in central Guizhou Province, southwestern China based on direct harvest method. *Acta Ecologica Sinica* 40, 4455–4461

Lu, S. Y., Pan, L. P., Peng, W. X., Song, T. Q., Du, H., and Liu, Y. X. (2018). Characteristics of biomass allocation in different bamboo plantations. *Ecological Science* 37, 123–129.

Luo, J., Dai, C. D., Tian, Y. X., Peng, P., Ma, F. F., Zeng, Z. Q., et al. (2016). Establishment of main constructive species biomass model for project forests of carbon sink in Hunan. *Hunan Forestry Science* & *Technology* 43, 12–21.

Peng, J. J., Wang, Z., Zhang, Y., Liu, H. Y., Gu, G. T., Peng, X. Y., et al. (2022). Construction of compatible individual tree biomass model of *Myrica rubra* plantation. *Journal of Zhejiang A&F University* 39, 272–279.

Sun, W. T., Zhou, J., and Li, L. H. (2022). Distribution characteristics of biomass and carbon density of *Pinus taiwanensis* hayata with different ages. *Journal of Shandong Agricultural University (Natural Science Edition)* 53, 469–474.

Xia, C. C., Yang, T. H., Cao, J., Zhou, Q., Lin, B., and Da, L. J. (2014). Biomass allocation and simulation of tree species in the shrub layer in Tiantong, Zhejiang Province. *Journal of Anhui Agricultural University*. 41, 945–949.

Xu, Wen., Hu, H. B., and Zhou, C. H. (2011). Biomass structure and distribution characters of *Pinus massoniana* in eastern region of Anhui Province in China. *Journal of Central South University of Forestry* & *Technology* 31, 111–115.

Yang, T. H., Da, L. J., Song, Y. C., Yang, Y. C., and Wang, L. Y. (2005). Biomass of evergreen broad-leaved forest in Tiantong National Forest ParkZhejiang Province. (Ⅰ) Community structure and fresh weight biomass of main tree species. *Journal of Zhejiang Forest College* 22, 363–369.

Yao, D. H., and Li, Z. H., (1997). Study on the biomass dynamics of *Cryptomeria japonica* plantation. *Scientia Silvae Sinicae* 33, 203–207.

Yu, M. J. (1999). Dynamics of an evergreen broadleaved forest dominated by *Cyclobalanopsis glauca* in southeast China. *Scientia Silvae* *Sinicae* 35, 42–51.

Zhang, J. (2016). Research on species diversity, biomass and carbon storage of different plantations in Guangxi karst area. Master’s thesis. Guangxi: Guangxi university.

Zhang, S., Xiang, W. H., Ou, Y. S., Zeng, Y. L., and Liu, C. (2015). Forest biomass and allocation pattern in 4 subtropical forests in hilly region of central Hunan Province. *Guangxi Forestry Science* 44, 104–109.

Zheng, H., Ouyang, Z. Y., Xu, W. H., Wang, X. K., Miao, H., Li, X. Q., et al. (2008). Variation of carbon storage by different reforestation types in the hilly red soil region of southern China. *Forest Ecology and Management* 255, 1113–1121.

Zuo, S. D., Ren, Y., Wang, X., Ding, H. F., Luo, and Y, J. (2015). Biomass allometric equations of nine common tree species in an evergreen broadleaved forest of subtropical China. *Chinese Journal of Applied Ecology* 26, 356–362.**Supplementary Table 3** Plant functional traits and their abbreviations, units, and calculations.

| Plant functional trait | Abbreviation | Unit | Calculation |
| --- | --- | --- | --- |
| Specific leaf area | SLA | cm^2^ g | Leaf area/dry mass |
| Leaf tissue density | LTD | g cm^-3^ | Leaf dry mass/volume |
| Leaf dry matter content | LDMC | g g^-1^ | Leaf dry mass/fresh mass |
| Twig tissue density | TTD | g cm^-3^ | Twig dry mass/volume |
| Twig dry matter content | TDMC | g g^-1^ | Twig dry mass/fresh mass |
| Bark tissue density | BTD | g cm^-3^ | Bark dry mass/volume |
| Bark dry matter content | BDMC | g g^-1^ | Bark dry mass/fresh mass |
| Stem tissue density | STD | g cm^-3^ | Stem dry mass/volume |
| Stem dry matter content | SDMC | g g^-1^ | Stem dry mass/fresh mass |

**Supplementary Table 4** Plant functional traits (mean ± SD) of overlapping species in conserved and disturbed forests in Zhejiang Province, east China.

| Species | Disturbance history | Leaf area (cm^2^) | Leaf thickness (mm) | Specific leaf area (cm^2^ g) | Leaf tissue density (g cm^-3^) | Leaf dry matter content (g g^-1^) | Twig tissue density (g cm^-3^) | Twig dry matter content (g g^-1^) | Bark thickness (mm) | Bark tissue density (g cm^-3^) | Bark dry matter content (g g^-1^) | Stem tissue density (g cm^-3^) | Stem dry matter content (g g^-1^) |
| --- | --- | --- | --- | --- | --- | --- | --- | --- | --- | --- | --- | --- | --- |
| *Cryptomeria japonica* var. *Sinensis* | Conserved forest | 0.19±0.03 | 0.66±0.12 | 78.65±7.36 | 0.20±0.03 | 0.42±0.04 | 0.51±0.10 | 0.48±0.03 | 0.40±0.15 | 0.29±0.04 | 0.40±0.03 | 0.31±0.03 | 0.33±0.04 |
|  | Disturbed forest | 0.21±0.03 | 1.02±0.09 | 73.67±12.29 | 0.14±0.02 | 0.47±0.03 | 0.58±0.04 | 0.52±0.04 | 0.35±0.07 | 0.30±0.01 | 0.44±0.03 | 0.34±0.04 | 0.33±0.02 |
| *Cunninghamia lanceolata* | Conserved forest | 1.10±0.37 | 0.59±0.05 | 79.27±18.80 | 0.22±0.04 | 0.37±0.05 | 0.31±0.06 | 0.39±0.03 | 1.48±0.33 | 0.25±0.04 | 0.49±0.02 | 0.33±0.02 | 0.32±0.01 |
|  | Disturbed forest | 1.17±0.31 | 0.55±0.12 | 59.77±6.43 | 0.32±0.06 | 0.46±0.03 | 0.47±0.03 | 0.44±0.02 | 0.96±0.43 | 0.19±0.02 | 0.49±0.05 | 0.33±0.03 | 0.35±0.05 |
| *Pinus massoniana* | Conserved forest | 1.23±0.59 | 0.77±0.06 | 83.01±31.89 | 0.18±0.07 | 0.39±0.05 | 0.45±0.02 | 0.44±0.04 | 1.30±0.28 | 0.30±0.03 | 0.67±0.12 | 0.39±0.05 | 0.44±0.04 |
|  | Disturbed forest | 0.90±0.44 | 0.75±0.23 | 57.29±11.06 | 0.25±0.05 | 0.44±0.03 | 0.48±0.04 | 0.45±0.02 | 1.29±0.48 | 0.32±0.03 | 0.73±0.06 | 0.39±0.07 | 0.41±0.10 |
| *Acer davidii* | Conserved forest | 40.90±12.53 | 0.32±0.05 | 236.44±56.07 | 0.14±0.03 | 0.31±0.04 | 0.39±0.02 | 0.54±0.01 | 0.29±0.07 | 0.44±0.02 | 0.45±0.02 | 0.47±0.02 | 0.57±0.04 |
|  | Disturbed forest | 37.01±5.68 | 0.29±0.08 | 180.80±28.15 | 0.20±0.03 | 0.37±0.01 | 0.46±0.02 | 0.56±0.01 | 0.20±0.10 | 0.40±0.04 | 0.40±0.04 | 0.49±0.01 | 0.53±0.09 |
| *Acer elegantulum* | Conserved forest | 30.66±4.92 | 0.27±0.03 | 245.10±22.90 | 0.15±0.02 | 0.38±0.03 | 0.38±0.04 | 0.52±0.01 | 0.16±0.01 | 0.50±0.02 | 0.51±0.01 | 0.51±0.04 | 0.52±0.04 |
|  | Disturbed forest | 27.59±6.73 | 0.30±0.03 | 225.65±46.09 | 0.16±0.03 | 0.42±0.05 | 0.50±0.04 | 0.56±0.02 | 0.22±0.05 | 0.61±0.07 | 0.58±0.02 | 0.59±0.06 | 0.57±0.08 |
| *Alniphyllum fortunei* | Conserved forest | 42.52±15.15 | 0.30±0.03 | 122.97±6.19 | 0.27±0.02 | 0.34±0.03 | 0.42±0.04 | 0.39±0.03 | 0.36±0.11 | 0.52±0.05 | 0.48±0.03 | 0.34±0.04 | 0.37±0.03 |
|  | Disturbed forest | 33.44±8.49 | 0.42±0.03 | 123.69±15.62 | 0.20±0.03 | 0.38±0.03 | 0.44±0.01 | 0.45±0.01 | 0.20±0.07 | 0.43±0.06 | 0.43±0.04 | 0.37±0.05 | 0.39±0.04 |
| *Castanopsis eyrei* | Conserved forest | 11.82±3.40 | 0.35±0.10 | 90.19±9.22 | 0.34±0.07 | 0.45±0.01 | 0.55±0.05 | 0.51±0.03 | 0.48±0.20 | 0.49±0.06 | 0.59±0.04 | 0.49±0.04 | 0.49±0.04 |
|  | Disturbed forest | 12.48±2.78 | 0.38±0.07 | 89.67±8.34 | 0.30±0.02 | 0.48±0.01 | 0.63±0.01 | 0.55±0.03 | 0.32±0.13 | 0.56±0.04 | 0.57±0.04 | 0.56±0.11 | 0.53±0.08 |
| *Celtis biondii* | Conserved forest | 14.31±1.90 | 0.30±0.06 | 130.07±20.56 | 0.27±0.06 | 0.36±0.04 | 0.49±0.07 | 0.52±0.08 | 0.13±0.04 | 0.60±0.05 | 0.60±0.04 | 0.58±0.03 | 0.60±0.03 |
|  | Disturbed forest | 14.95±1.24 | 0.36±0.03 | 102.33±11.12 | 0.28±0.02 | 0.41±0.03 | 0.61±0.04 | 0.63±0.05 | 0.28±0.18 | 0.87±0.11 | 0.68±0.02 | 0.64±0.07 | 0.64±0.05 |
| *Choerospondias axillaris* | Conserved forest | 21.07±1.84 | 0.23±0.08 | 178.15±25.83 | 0.28±0.09 | 0.36±0.02 | 0.38±0.04 | 0.44±0.20 | 0.74±0.24 | 0.37±0.02 | 0.43±0.02 | 0.45±0.03 | 0.46±0.05 |
|  | Disturbed forest | 20.16±2.14 | 0.37±0.05 | 158.08±18.08 | 0.17±0.03 | 0.39±0.02 | 0.38±0.04 | 0.46±0.06 | 0.56±0.15 | 0.37±0.05 | 0.41±0.07 | 0.43±0.10 | 0.41±0.10 |
| *Cornus hongkongensis* subsp. *elegans* | Conserved forest | 16.64±6.35 | 0.26±0.02 | 121.42±12.70 | 0.32±0.02 | 0.35±0.01 | 0.56±0.04 | 0.52±0.02 | 0.28±0.05 | 0.63±0.04 | 0.58±0.02 | 0.59±0.04 | 0.54±0.04 |
|  | Disturbed forest | 18.47±3.75 | 0.43±0.05 | 99.73±7.05 | 0.24±0.04 | 0.38±0.01 | 0.58±0.01 | 0.52±0.03 | 0.18±0.08 | 0.68±0.12 | 0.60±0.09 | 0.79±0.25 | 0.58±0.05 |
| *Machilus thunbergii* | Conserved forest | 16.94±2.11 | 0.27±0.03 | 82.79±19.91 | 0.47±0.05 | 0.47±0.04 | 0.52±0.04 | 0.49±0.03 | 0.35±0.10 | 0.46±0.05 | 0.45±0.02 | 0.52±0.03 | 0.52±0.07 |
|  | Disturbed forest | 14.37±3.75 | 0.39±0.04 | 67.32±7.33 | 0.39±0.03 | 0.53±0.02 | 0.55±0.02 | 0.59±0.02 | 0.66±0.13 | 0.53±0.03 | 0.55±0.03 | 0.55±0.03 | 0.57±0.02 |
| *Phoebe sheareri* | Conserved forest | 52.74±19.17 | 0.28±0.03 | 140.63±11.70 | 0.26±0.02 | 0.39±0.02 | 0.48±0.02 | 0.45±0.03 | 0.29±0.03 | 0.54±0.06 | 0.50±0.02 | 0.48±0.07 | 0.52±0.05 |
|  | Disturbed forest | 44.23±6.49 | 0.44±0.05 | 126.25±21.54 | 0.19±0.04 | 0.44±0.04 | 0.50±0.04 | 0.49±0.04 | 0.36±0.11 | 0.60±0.05 | 0.60±0.02 | 0.58±0.03 | 0.61±0.02 |
| *Prunus schneideriana* | Conserved forest | 24.79±3.82 | 0.30±0.08 | 147.74±19.84 | 0.24±0.07 | 0.41±0.04 | 0.53±0.05 | 0.54±0.04 | 0.32±0.14 | 0.52±0.05 | 0.57±0.07 | 0.53±0.07 | 0.54±0.06 |
|  | Disturbed forest | 19.06±5.63 | 0.38±0.09 | 171.28±29.07 | 0.16±0.04 | 0.39±0.04 | 0.68±0.08 | 0.58±0.08 | 0.18±0.07 | 0.68±0.13 | 0.66±0.07 | 0.66±0.04 | 0.65±0.03 |
| *Quercus glauca* | Conserved forest | 34.34±10.50 | 0.28±0.03 | 84.49±6.07 | 0.43±0.05 | 0.49±0.02 | 0.65±0.03 | 0.52±0.01 | 0.30±0.09 | 0.62±0.03 | 0.59±0.01 | 0.68±0.05 | 0.57±0.02 |
|  | Disturbed forest | 28.18±2.21 | 0.50±0.07 | 76.99±6.38 | 0.27±0.02 | 0.55±0.01 | 0.66±0.03 | 0.57±0.02 | 0.56±0.35 | 0.70±0.04 | 0.64±0.03 | 0.71±0.04 | 0.63±0.05 |
| *Schima superba* | Conserved forest | 35.16±10.97 | 0.25±0.03 | 109.76±8.37 | 0.36±0.02 | 0.37±0.02 | 0.47±0.04 | 0.42±0.01 | 0.39±0.09 | 0.46±0.04 | 0.50±0.01 | 0.57±0.04 | 0.55±0.02 |
|  | Disturbed forest | 25.37±7.19 | 0.47±0.03 | 81.42±4.65 | 0.26±0.03 | 0.42±0.01 | 0.57±0.01 | 0.52±0.02 | 0.55±0.08 | 0.60±0.03 | 0.61±0.03 | 0.52±0.04 | 0.48±0.07 |
| *Eurya muricata* | Conserved forest | 18.43±3.01 | 0.41±0.02 | 79.98±14.81 | 0.31±0.04 | 0.35±0.03 | 0.59±0.04 | 0.49±0.02 | 0.26±0.04 | 0.58±0.07 | 0.53±0.03 | 0.54±0.05 | 0.51±0.04 |
|  | Disturbed forest | 13.40±1.55 | 0.56±0.06 | 69.76±4.13 | 0.26±0.01 | 0.41±0.01 | 0.62±0.06 | 0.56±0.01 | 0.13±0.03 | 0.63±0.06 | 0.61±0.03 | 0.72±0.03 | 0.64±0.03 |
| *Itea omeiensis* | Conserved forest | 32.13±7.98 | 0.29±0.02 | 106.89±10.72 | 0.33±0.03 | 0.35±0.01 | 0.46±0.04 | 0.46±0.03 | 0.35±0.11 | 0.52±0.06 | 0.53±0.06 | 0.67±0.03 | 0.61±0.02 |
|  | Disturbed forest | 22.81±5.40 | 0.48±0.03 | 105.54±15.26 | 0.20±0.02 | 0.34±0.03 | 0.46±0.05 | 0.50±0.04 | 0.22±0.07 | 0.52±0.12 | 0.53±0.08 | 0.66±0.07 | 0.64±0.02 |
| *Lindera erythrocarpa* | Conserved forest | 24.49±6.07 | 0.22±0.04 | 171.66±28.59 | 0.28±0.06 | 0.32±0.04 | 0.42±0.05 | 0.47±0.05 | 0.29±0.09 | 0.40±0.04 | 0.41±0.02 | 0.49±0.05 | 0.49±0.04 |
|  | Disturbed forest | 22.60±7.39 | 0.34±0.03 | 160.30±34.74 | 0.19±0.04 | 0.38±0.04 | 0.47±0.02 | 0.58±0.03 | 0.27±0.08 | 0.47±0.04 | 0.47±0.01 | 0.58±0.03 | 0.57±0.02 |
| *Loropetalum chinense* | Conserved forest | 5.60±1.52 | 0.30±0.04 | 126.06±15.61 | 0.27±0.03 | 0.38±0.02 | 0.58±0.03 | 0.45±0.05 | 0.16±0.03 | 0.39±0.05 | 0.43±0.03 | 0.69±0.04 | 0.58±0.03 |
|  | Disturbed forest | 4.70±1.24 | 0.37±0.04 | 118.49±6.76 | 0.23±0.02 | 0.42±0.01 | 0.64±0.02 | 0.54±0.03 | 0.17±0.03 | 0.50±0.06 | 0.53±0.03 | 0.74±0.04 | 0.64±0.03 |
